# Correction to ‘The kinase inhibitor palbociclib binds to HIV TAR RNA with very low nanomolar affinity and exquisite specificity’

**DOI:** 10.1093/nar/gkag774

**Published:** 2026-07-30

This is a correction to: Ravikanth Reddy Ramireddy, Matthew D Shortridge, Venkata Vidalala, Bhawna Chaubey, Changyan Tang, Gregory L Olsen, Gabriele Varani, The kinase inhibitor palbociclib binds to HIV TAR RNA with very low nanomolar affinity and exquisite specificity, *Nucleic Acids Research*, Volume 54, Issue 5, 24 March 2026, gkag186, https://doi.org/10.1093/nar/gkag186

In the originally published version of this manuscript, there was a typographical error in the first author’s name. This has been corrected to read “Ravikanth Reddy Ramireddy”.

